# NGF Modulates Cholesterol Metabolism and Stimulates ApoE Secretion in Glial Cells Conferring Neuroprotection against Oxidative Stress

**DOI:** 10.3390/ijms23094842

**Published:** 2022-04-27

**Authors:** Mayra Colardo, Michele Petraroia, Letizia Lerza, Daniele Pensabene, Noemi Martella, Valentina Pallottini, Marco Segatto

**Affiliations:** 1 Department of Biosciences and Territory, University of Molise, Contrada Fonte Lappone, 86090 Pesche, Italy; m.colardo@studenti.unimol.it (M.C.); m.petraroia2@studenti.unimol.it (M.P.); l.lerza@studenti.unimol.it (L.L.); n.martella@studenti.unimol.it (N.M.); 2 Department of Science, University Roma Tre, Viale Marconi 446, 00146 Rome, Italy; dan.pensabene@stud.uniroma3.it (D.P.); valentina.pallottini@uniroma3.it (V.P.); 3 Neuroendocrinology Metabolism and Neuropharmacology Unit, IRCSS Fondazione Santa Lucia, Via del Fosso Fiorano 64, 00143 Rome, Italy

**Keywords:** ApoE, astrocyte, cholesterol, differentiation, nerve growth factor, metabolism, neuron, reactive oxygen species, rotenone

## Abstract

Cholesterol plays a crucial role in the brain, where its metabolism is particularly regulated by astrocytic activity. Indeed, adult neurons suppress their own cholesterol biosynthesis and import this sterol through ApoE-rich particles secreted from astrocytes. Recent evidence suggests that nerve growth factor (NGF) may exert neurotrophic activity by influencing cell metabolism. Nevertheless, the effect of NGF on glial cholesterol homeostasis has still not been elucidated. Thus, the aim of this project is to assess whether NGF could influence cholesterol metabolism in glial cells. To reach this objective, the U373 astrocyte-derived cell line was used as an experimental model. Immunoblot and ELISA analysis showed that proteins and enzymes belonging to the cholesterol metabolism network were increased upon NGF treatment in glial cells. Furthermore, NGF significantly increased ApoE secretion and the amount of extracellular cholesterol in the culture medium. Co-culture and U373-conditioned medium experiments demonstrated that NGF treatment efficiently counteracted rotenone-mediated cytotoxicity in N1E-115 neuronal cells. Conversely, neuroprotection mediated by NGF treatment was suppressed when N1E-115 were co-cultured with ApoE-silenced U373 cells. Taken together, these data suggest that NGF controls cholesterol homeostasis in glial cells. More importantly, NGF exerts neuroprotection against oxidative stress, which is likely associated with the induction of glial ApoE secretion.

## 1. Introduction

The brain is characterized by the highest amount of cholesterol in the whole body [1]. The exceptional concentration of this sterol in the central nervous system (CNS) is justified by the fact that cholesterol is involved in several fundamental biological processes. Of note, a significant amount of cholesterol is integrated into plasma membranes of astrocytes and neurons, where it participates in defining a proper cellular morphology, as well as the correct functioning of membrane receptors and synaptic neurotransmission [2]. Cholesterol is also essential to form new membranes required for neurite outgrowth, synaptogenesis, and neurotransmitter release, thus exerting a crucial role in neuronal differentiation and maturation [3,4,5,6,7].

Essentially, all cholesterol in the brain derives from de novo biosynthesis in situ. Specifically, the blood brain barrier (BBB) prevents cholesterol uptake deriving from lipoproteins transported through the systemic circulation [8]. For this reason, nerve cells develop a fine regulatory network to independently manage their own demand for this lipid. A well-accepted model establishes that after embryogenesis, during the postnatal period, cholesterol biosynthesis dramatically drops in neurons, with the attempt to save the energy employed for the generation of the action potentials. Accordingly, astrocytes satisfy neuronal requirements of cholesterol, whose synthesis becomes particularly pronounced [1,9,10]. The delivery of cholesterol produced by astrocytes to neurons implies an elegant mechanism of horizontal transport, which involves key proteins and particles. Specifically, cholesterol export starts in astrocytes, where neo-synthetized cholesterol is loaded into nascent lipoprotein particles containing apolipoprotein E (ApoE). Accumulating evidence indicates that the lipidation of ApoE-rich particles is mediated by the transporter ATP-binding cassette A1 (ABCA1) [11]. Subsequently, once released into the extracellular space, ApoE-containing lipoproteins are up taken by neurons through receptor-mediated endocytosis via low density lipoprotein receptor (LDLr) and LDLr-related protein 1 (LRP1) [12]. According to the pivotal role of astrocyte-derived cholesterol in neuronal homeostasis, experimental findings indicated that cholesterol contained in ApoE-rich lipoproteins significantly enhances dendrite differentiation and synaptic function [13,14]. Accordingly, astrocyte-conditioned medium from Huntington’s disease (HD) astrocytes, which only secrete a modest amount of ApoE-lipoprotein-bound cholesterol, severely affects neurite outgrowth and synaptic activity in HD neurons [15]. The neurotrophic action of astrocyte-derived ApoE-containing particles is further sustained by the impact of ApoE deficiency, which markedly hampers adult neurogenesis in the hippocampus [16]. Astrocyte–neuron cholesterol crosstalk is also critical in neuroprotection against oxidative stress. For instance, some reports show that ApoE hinders oxidative damage and apoptosis induced by hydrogen peroxide exposure [17,18].

From these observations, the maintenance of cholesterol homeostasis in the brain is necessary to guarantee the functioning of nerve cells, and the metabolic pathways underlying the regulation of this lipid should be finely controlled to preserve the occurrence of several neurophysiological processes.

In recent years, neurotrophins have emerged as important players in cell metabolism [2,19,20,21,22]. Neurotrophins belong to a small family of growth factors that have an incontrovertible role in neuronal development, survival, and plasticity [23]. More recently, these signaling molecules have also been recognized as crucial modulators of cell metabolism, including cholesterol metabolism [2,22].

Experimental evidence highlights that nerve growth factor (NGF), the prototype member of the neurotrophin family, stimulates cholesterol biosynthesis in neuronal cells and hepatocytes [24,25]. Interestingly, these events involve, at least in part, the low-affinity neurotrophin receptor p75NTR, which elicits activation of the sterol regulatory element binding protein 2 (SREBP-2) and, in turn, induces the transcription of cholesterol-related genes [25]. The activity of NGF on cholesterol metabolism is not restricted to biosynthesis, as it also promotes cholesterol uptake by increasing LDLr expression in neurons [26].

Despite this information, knowledge about the possible contribution of NGF in the regulation of cholesterol homeostasis still appears particularly limited. Notably, there is a lack of systematic studies assessing the role played by NGF in the regulation of cholesterol metabolism in astrocytic cells. Considering that cholesterol produced by astrocytes is essential to guarantee neuronal physiology, it is important to understand whether NGF is able to influence cholesterol metabolism in glial cells as well as intercellular cholesterol transport, whose peculiar regulation is imperative to assure brain health. 

On this basis, the aim of this work is to explore the prospective involvement of NGF in the modulation of cholesterol metabolism in glial cells, as well as its impact on neuronal differentiation and neuronal survival under oxidative stress conditions.

## 2. Results

### 2.1. U373 Express Both the NGF Receptors TrkA and p75NTR

NGF was initially identified as a growth factor with a specific tropism for neuronal cells. However, accumulating evidence clearly demonstrate that this neurotrophin also induces biological effects in several non-neuronal cells and tissues [22]. The activity of NGF is assured by its binding to the high-affinity receptor TrkA and to the low-affinity receptor p75NTR [2]. Thus, we initially ascertained whether U373 cells express NGF receptors. Notably, U373 is an astrocytoma cell line widely employed as an astrocyte cell culture model, as several reports indicated that they reproduce a number of morphological, metabolic, and functional properties of astrocytes [27,28,29]. According to published data [30], immunofluorescence analysis showed that U373 cells express both TrkA and p75NTR at appreciable levels (Figure S1A). These results were also confirmed by Western blot analysis, suggesting that U373 cells have the potential to be fully responsive to NGF. Since the administration of NGF may affect the expression levels of its receptors, potentially altering the balance between signaling pathways elicited by TrkA and/or p75NTR [31,32,33], the expression of both receptors was also checked upon 48 h of NGF treatment. However, no changes were observed in the protein amount of both TrkA and p75NTR after the stimulation with the neurotrophin (Figure S1B).

### 2.2. NGF Modulates the Protein Network Involved in Cholesterol Homeostasis in Glial Cells

**Figure 1:**
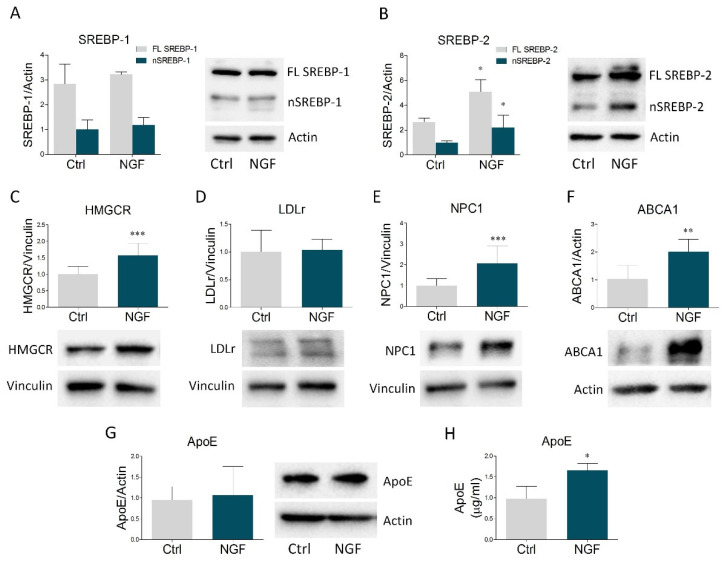
NGF modulates the expression of the main proteins involved in cholesterol homeostasis in U373 cells and enhances ApoE secretion in U373 culture medium. (**A**–**G**) Representative Western blot and densitometric analysis of SREBP-1, SREBP-2, HMGCR, LDLr, NPC1, ABCA1, and ApoE in U373 cells treated with vehicle (Ctrl) and NGF (100 ng/mL) for 48 h. *n* = 6 different experiments. Actin and vinculin were used as loading controls. (**H**) Quantification of ApoE levels (µg/mL) by ELISA assay in vehicle- and NGF-treated U373 culture medium. *n* = 3 different experiments. Data represent means ± SD. Statistical analysis was assessed by using unpaired Student’s *t* test. * *p* < 0.05, ** *p* < 0.01, *** *p* < 0.001.

Once ascertained that U373 cells are responsive to NGF, we evaluated the putative role of NGF in the modulation of the main proteins belonging to cholesterol homeostasis machinery. We started to check SREBP-1 and SREBP-2, the most relevant transcription factors of lipid-related genes. In particular, when intracellular lipids are low, SREBPs are activated by proteolytic cleavage, generating a transcriptionally-active fragment that enters the nucleus and induces the transcription of target genes [2]. Immunoblot analysis revealed that NGF treatment did not affect the expression of the full length and the nuclear form of SREBP-1 (Figure 1A). Conversely, stimulation with the neurotrophin significantly increased both the full length and the active fraction of SREBP-2 (Figure 1B). We next evaluated HMGCR expression, the key and rate-limiting enzyme of cholesterol biosynthesis, revealing a build-up of this enzyme following NGF treatment (Figure 1C). While LDLr was not affected (Figure 1D), NGF treatment increased NPC1, which is involved in intracellular cholesterol trafficking (Figure 1E). In addition, NGF significantly raised ABCA1 levels (Figure 1F), the most important transporter involved in cholesterol efflux from astrocytes [2], whereas intracellular ApoE expression did not show any variation. Interestingly, the ELISA assay displayed a significant ApoE enrichment in U373 conditioned medium upon NGF exposure (Figure 1H). Taken together, these results indicate that NGF markedly impacts cholesterol metabolism in glial cells by increasing the expression of proteins involved in cholesterol biosynthesis, trafficking, and efflux. Of note, the induction of ABCA1 expression strongly suggests an enhancement in the secretion of ApoE-rich particles from astrocytes, which is corroborated by the rise in ApoE levels into the culture medium.

### 2.3. NGF Increases Cholesterol Secretion by U373 into the Culture Medium

**Figure 2:**
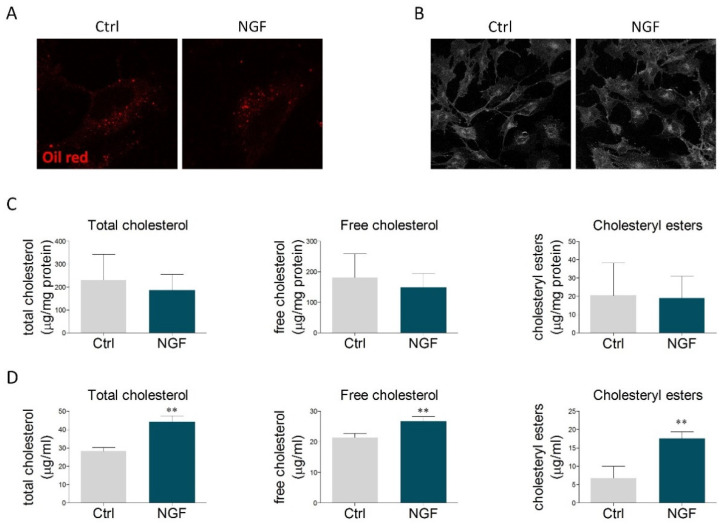
NGF effects on neutral lipids and cholesterol content. (**A**) U373 cells were treated with vehicle (Ctrl) or NGF (100 ng/mL) for 48 h and then stained with Oil Red O to visualize the intracellular content of neutral lipids. *n* = 3 different experiments. (**B**) Representative image of filipin staining performed on U373 cells treated with vehicle (Ctrl) and NGF (100 ng/mL) for 48 h. *n* = 3 different experiments. (**C**) Quantification of intracellular cholesterol levels (total cholesterol, free cholesterol, and cholesteryl esters) in vehicle- and NGF-treated U373 cells. *n* = 3 different experiments. (**D**) Cholesterol quantification (total cholesterol, free cholesterol, and cholesteryl esters) in the culture medium of U373 cells treated with vehicle (Ctrl) and NGF (100 ng/mL) for 48 h. *n* = 3 different experiments. Data represent means ± SD. Statistical analysis was performed by using unpaired Student’s *t*-test. ** *p* <0.01.

The obtained results prompted us to study whether the effects mediated by NGF could reflect the modulation of cholesterol content. Oil Red O staining was performed to visualize neutral lipids, including cholesteryl esters. The size and the number of lipid droplets found in U373 cells were similar to those ones reported for primary astrocytes [34,35]. However, no alterations were observed upon NGF treatment (Figure 2A and Figure S2A,B). Similarly, no changes were detected in the amount of free cholesterol, evaluated by filipin staining (Figure 2B and Figure S2C). The lack of significant effects on intracellular cholesterol levels was further sustained by cholesterol enzymatic assay, which highlighted that NGF did not alter the content of total cholesterol, free cholesterol, and cholesteryl esters (Figure 2C). Conversely, the amount of cholesterol (free, esters, and total) was markedly increased in the culture medium of U373-treated NGF (Figure 2D). When evaluated as a whole, these data indicate that NGF promotes cholesterol extrusion by enhancing ABCA1-mediated efflux of ApoE-containing particles.

### 2.4. p75NTR Is Partially Involved in the NGF-Mediated Effects on Cholesterol Protein Network

**Figure 3:**
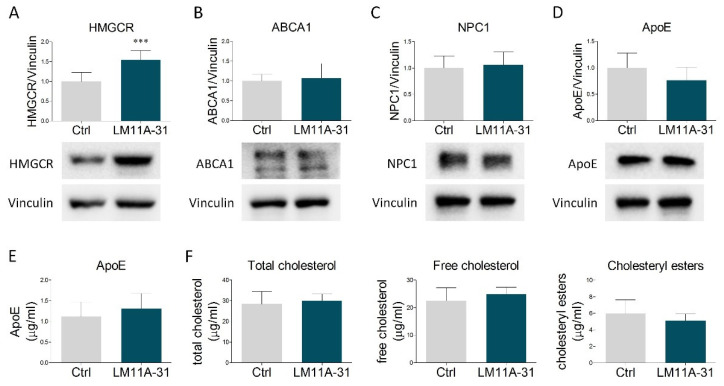
Effects of p75NTR modulation by LM11A-31 on cholesterol metabolism and secretion. (**A**–**D**) Representative Western blot and densitometric analysis of HMGCR, ABCA1, NPC1, and ApoE in U373 cells treated with vehicle (Ctrl) and LM11A-31 (0.1 µM) for 48 h. *n* = 4 different experiments. Vinculin served as a housekeeping protein to normalize protein loading. (**E**) Quantification of ApoE amount (µg/mL) by ELISA assay in culture medium from vehicle- and LM11A-31-treated U373 cells. *n* = 3 different experiments. (**F**) Cholesterol quantification (total cholesterol, free cholesterol, and cholesteryl esters) in the culture medium of U373 cells treated with vehicle (Ctrl) and LM11A-31 (0.1 µM) for 48 h. *n* = 3 different experiments. Data represent means ± SD. Statistical analysis was carried out by using unpaired Student’s *t* test. *** *p* < 0.001.

Experimental findings show that NGF affects cholesterol metabolism in liver cells through the activation of its low-affinity receptor p75NTR [25]. To test whether this signaling pathway may be also preserved in astrocytic cells, we treated U373 with LM11A-31, a small ligand acting as a specific p75NTR modulator. This compound does not activate TrkA but, similarly to NGF, elicits the induction of p75NTR downstream signaling pathways [36]. LM11A-31 mimicked the NGF-mediated increase of HMGCR (Figure 3A). However, no changes were observed in the other proteins involved in cholesterol metabolism (Figure 3B–D), nor in the amount of extracellular ApoE (Figure 3E) and cholesterol (total, free and esters) released into the culture medium (Figure 3F). Collectively, these results indicate that NGF-induced expression of HMGCR is mediated by p75NTR activity, whereas the effects on NPC1, ABCA1, ApoE secretion, and cholesterol extrusion are likely dependent on other transduction pathways, possibly involving TrkA. 

### 2.5. Conditioned Medium Derived from NGF-Treated U373 Cells Does Not Influence Neuronal Differentiation

**Figure 4:**
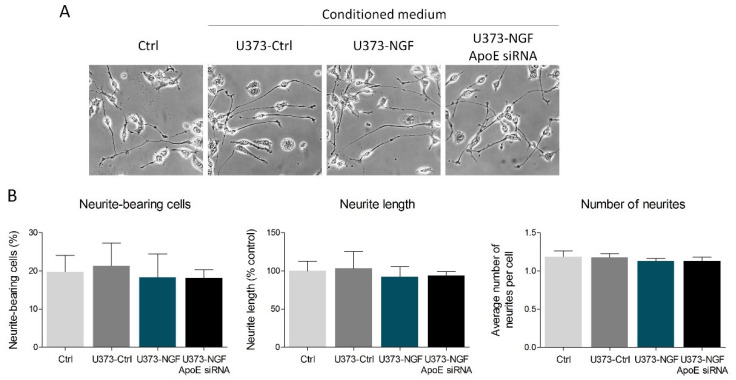
Effect of U373 conditioned medium on neuronal differentiation. (**A**) Representative images in bright field of N1E-115 cells cultured in fresh DMEM (Ctrl), in conditioned medium derived from control U373 (U373-Ctrl), NGF-pre-treated U373 (U373-NGF), and NGF-pre-treated U373 silenced for ApoE (U373-NGF ApoE siRNA) for 48 h. (**B**) Morphological analysis of neurite-bearing cells, neurite length and number of neurites of N1E-115 cells in the presence of fresh DMEM (Ctrl), in conditioned medium derived from control U373 (U373-Ctrl), NGF-pre-treated U373 (U373-NGF), and NGF-pre-treated U373 silenced for ApoE (U373-NGF ApoE siRNA) for 48 h. *n* = 5 different experiments. Data represent means ± SD. Statistical analysis was performed by using one-way ANOVA, followed by Tukey’s post hoc test.

Published data demonstrate that astrocyte-derived ApoE particles could impact neurite outgrowth in neurons [15]. Thus, we evaluated whether NGF-mediated secretion of ApoE can affect neuritogenesis in the neuron-like cell line N1E-115. To reach this aim, N1E-115 were induced to differentiate for 48 h in the presence of conditioned medium derived from control U373 (U373-Ctrl), NGF-pre-treated U373 (U373-NGF), and NGF-pre-treated U373 silenced for ApoE (U373-NGF ApoE siRNA). The obtained results showed that no effects were detectable in the percentage of neurite-bearing cells, neurite length and number of neurites among the experimental groups taken into consideration in this study (Figure 4A,B). Therefore, U373-conditioned medium and/or U373-derived ApoE do not influence N1E-115 neuritogenesis, even when U373 are pre-treated with NGF.

### 2.6. NGF-Mediated Secretion of ApoE by U373 Protects Neuronal Cells from Oxidative Insult

**Figure 5:**
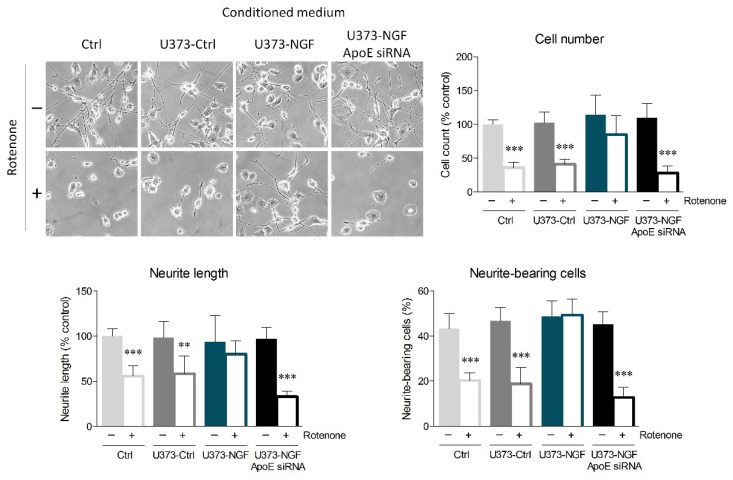
Effects of U373 conditioned medium on oxidative stress in neurons. Representative images in bright field and quantitative assessment of neuronal morphology of N1E-115. Cells were previously treated (+) or not (−) with rotenone (0.1 µM) for 16 h, and then cultured in fresh DMEM (Ctrl), in conditioned medium derived from control U373 (U373-Ctrl), NGF-pre-treated U373 (U373-NGF), and NGF-pre-treated U373 silenced for apoE (U373-NGF ApoE siRNA) for 48 h. *n* = 5 different experiments. Data represent means ± SD. Statistical analysis was assessed by using one-way ANOVA, followed by Tukey’s post hoc test. ** *p* < 0.01, *** *p* < 0.001.

Accumulating evidence highlights that ApoE increases neuronal resilience to oxidative insults, thus preventing apoptosis and neurodegeneration [17,18,37]. In this context, we evaluated whether NGF-induced ApoE secretion by U373 cells could protect N1E-115 neurons from oxidative stress. Fully differentiated N1E-115 were pre-treated with rotenone, which induces oxidative stress by inhibiting the mitochondrial complex I. After 16 h of rotenone exposure, N1E-115 neurons were cultured in fresh medium (Ctrl), conditioned medium from U373 cells (U373-Ctrl), conditioned medium from U373 pre-treated with NGF for 48 h (U373-NGF), or conditioned medium from ApoE-silenced U373 pre-treated with NGF for 48 h (U373-NGF ApoE siRNA). Rotenone pre-treatment strongly decreased the number of N1E-115 cultured in control medium, indicating that the administration of this pesticide severely impacts N1E-115 survival. Rotenone cytotoxicity also determined intense neurite retraction, as observed by the length of neurites. Neurite damage elicited by rotenone treatment was particularly evident upon evaluation of neurite-bearing cells, as the number of N1E-115 with neurites decreased to 50%. Similar results were obtained when N1E-115 cells were cultured in U373-Ctrl conditioned medium, suggesting that the presence of supernatant derived from unstimulated astrocytes is not sufficient to protect neuronal cells from rotenone-mediated toxicity. On the contrary, application of conditioned medium derived from NGF-treated U373 effectively prevented neuronal death, as well as the reduction in neurite-bearing cells and neurite length caused by rotenone administration (Figure 5). More importantly, conditioned medium derived from ApoE-silenced U373 cells completely lost the beneficial effects induced by U373-NGF conditioned medium, indicating that neuroprotection is driven by the NGF-mediated ApoE release in the culture medium. Notably, conditioned medium from NGF-treated U373 transfected with scrambled siRNA fully retained the protective effects exerted by U373-NGF conditioned medium, demonstrating that the loss of neuroprotection observed in conditioned medium from ApoE-silenced cells is effectively dependent upon ApoE downregulation (Figure S3C).

**Figure 6:**
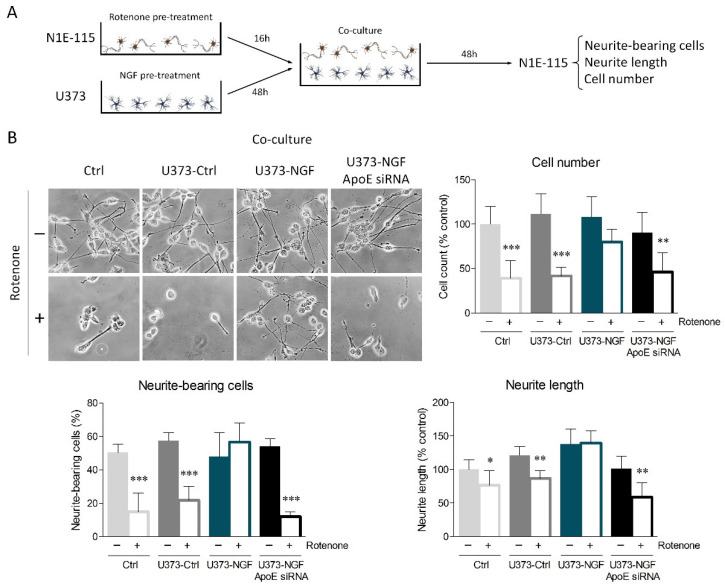
ApoE secretion by NGF-treated U373 cells provides neuroprotection from oxidative stress. (**A**) Representative scheme of astrocyte–neuron co-cultures set up as described in the Materials and Methods Section. Neurite-bearing cells, neurite length, and cell number were assessed 48 h after the establishment of co-culture. (**B**) Representative images in bright field and quantitative assessment of neuronal morphology of N1E-115 cells, previously treated (+) or not (−) with rotenone (0.1 µM) for 16 h. N1E-115 were then kept in fresh DMEM (Ctrl), or co-cultured with control U373 (U373-Ctrl), NGF-pre-treated U373 (U373-NGF), and NGF-pre-treated U373 silenced for ApoE (U373-NGF ApoE siRNA) for 48 h. *n* = 5 different experiments. Data represent means ± SD. Statistical analysis was carried out by using one-way ANOVA, followed by Tukey’s post hoc test. * *p* < 0.05, ** *p* < 0.01, *** *p* < 0.001.

To strengthen the results obtained from conditioned medium, we performed U373/N1E-115 co-cultures. To start, U373 cells were pre-treated or not with NGF for 48 h. Furthermore, differentiated N1E-115 cells were pre-treated or not with rotenone for 16 h. Subsequently, U373 medium was replaced with fresh medium containing 0.5% FBS, and coverslips seeded with N1E-115 were transferred onto the U373 layer to prepare co-cultures (Figure 6A). Oxidative damage elicited by rotenone treatment considerably affected cell viability, neurite-bearing cells, and neurite length of control N1E-115 and N1E-115 co-cultured with U373-Ctrl astrocytes (Figure 6B). Consistent with conditioned medium experiments, N1E-115 cells co-cultured with U373-NGF cells were protected from oxidative damage, whereas ApoE silencing in U373 abolished the neuroprotection sustained by NGF.

## 3. Discussion

Imbalances in neurotrophin signaling pathways, as well as alterations in brain cholesterol metabolism, are strongly associated with neurologic and neurodegenerative diseases, such as Rett syndrome, HD, Alzheimer’s disease (AD), and Parkinson’s disease (PD) [2,38,39]. In this context, fundamental research on the biological activity of neurotrophins is pivotal to better dissect the molecular mechanisms linking cholesterol metabolism and brain physiopathology. During the last few years, an interesting study reported a role for BDNF in the regulation of cholesterol metabolism in astrocytes [38]. Despite this notion, no information is available about the putative involvement of NGF in the regulation of astrocytic cholesterol. Therefore, in this work we focused on the prospective role exerted by NGF in the regulation of cholesterol metabolism, with particular reference to the influence of ApoE secretion on neuronal differentiation and survival. U373, the astrocytoma cell line used as an experimental model of human astrocytes, retains the potential to fully respond to NGF, as it expresses appreciable levels of both TrkA and p75NTR. The main results indicated that NGF upregulates the levels of the main proteins involved in cholesterol biosynthesis (HMGCR), intracellular trafficking (NPC1), and secretion (ABCA1). These events are accompanied by a concurrent increase of ApoE and cholesterol levels into the culture medium, suggesting that NGF increases cholesterol biosynthesis and extrusion from glial cells. It has been reported that NGF induces ApoE protein expression by increasing its transcription [40]. However, we did not observe any significant change in intracellular ApoE expression. This apparent inconsistency can be explained since ApoE buildup cannot be appreciated because of its increased efflux from NGF-treated U373. With the attempt to better dissect the molecular mechanisms, we treated U373 with the LM11A-31 and found that the p75NTR modulator was only able to mimic the rise in HMGCR expression mediated by NGF. The involvement of p75NTR in the modulation of HMGCR levels is consistent with previous data obtained in other cell types, showing that this receptor controls the transcription of cholesterogenic enzymes [41]. However, LM11A-31 did not induce any changes either in the other proteins analyzed in this study, including ABCA1, or in cholesterol and ApoE levels into the culture medium, indicating that the selective modulation of p75NTR is not sufficient to promote cholesterol extrusion through ApoE-rich particles observed in NGF-treated cells. These results lead us to hypothesize that NGF governs cholesterol metabolism in glial cells through the involvement of both p75NTR and TrkA receptors. It is well known that neurotrophin binding to Trk receptors activates ERK1/2, thus regulating several pathways involved in cell differentiation, proliferation, and survival [2,38]. Interestingly, it has been observed that ERK1/2 activation also regulates ABCA1 and ApoE expression in different cell types [38,42,43]. These findings, together with the evidence that the BDNF/TrkB/ERK axis promotes ApoE extrusion and ABCA1 expression in glial cells [38], suggest that a signal transduction pathway involving the TrkA/ERK axis may explain a part of the effects exerted by NGF in our study. 

While no changes were observed in neurite outgrowth, the employment of U373-conditioned medium and the establishment of co-culture experiments indicated that the increased ApoE secretion from glial cells mediated by NGF is determinant to prevent the detrimental outcomes induced by oxidative stress in neuronal cells. To induce cytotoxicity, we employed rotenone, a pesticide commonly used in preclinical research to induce mitochondria-derived reactive oxygen species (ROS) and subsequent apoptosis [44]. Our results highlight that NGF stimulates ApoE secretion by glial cells, which is necessary to guarantee the neuroprotective effects against rotenone administration in differentiated N1E-115 cells. The role of ApoE in the prevention of oxidative stress is well-documented. For instance, ApoE depletion in mouse models exacerbates oxidative injury in brain tissue [45,46]. Accordingly, administration of exogenous ApoE protects against irreversible oxidative damage from hydrogen peroxide treatment by counteracting secondary glutamate toxicity [18]. In addition, ApoE-containing lipoproteins are effective in attenuating apoptosis induced by withdrawal of trophic support in retinal ganglion neurons [47]. In our study, the employment of rotenone suggests that ApoE-mediated neuroprotection, promoted by NGF-stimulated glial cells, may be relevant in the context of neurodegenerative conditions such as PD. Notably, rotenone reproduces dopaminergic neuronal degeneration similar to that observed in PD, both in animal models and cell cultures, including N1E-115-derived neurons [48,49,50]. Coherently, our results support previous evidence showing that ApoE elicits neuroprotection upon the administration of 6-hydroxydopamine (6-OHDA), another neurotoxin capable of inducing a PD-like phenotype [51]. Collectively, our study demonstrates that NGF is a crucial modulator of cholesterol metabolism and ApoE extrusion from glial cells. Most importantly, the enhancement in ApoE secretion promoted by this neurotrophin is required to assure neuroprotection against rotenone cytotoxicity. BDNF is the most abundant neurotrophin in the adult brain. Despite this evidence, it has been shown that NGF may play a crucial role not only during development, but also in the adult central nervous system. For instance, NGF influences hippocampal activity and, as a consequence, spatial memory and learning [52]. Other findings suggest that NGF exhibits a neuroprotective effect on nigrostriatal dopaminergic neurons, and that this activity may set the basis for novel NGF-based therapeutic approaches for PD [53]. According to this notion, it has been shown that the production of NGF is deeply regulated in astrocytes during adulthood and exerts modulatory actions on neuroinflammation, brain injury, and neurodegeneration [54,55,56]. In this context, it would be interesting to evaluate, in future studies, whether these effects could be mediated, at least in part, by the modulation of glial cholesterol promoted by NGF. The present work presents some limitations that should be overcome in the next experimental investigations; for instance, it would be important to better dissect the molecular mechanisms linking NGF treatment and cholesterol metabolism in glial cells. A broad understanding is useful to identify specific molecular targets amenable to pharmacological manipulation that could be employed in neurodegenerative contexts. Furthermore, although U373 and N1E-115 cells are widely used as manageable cell lines to respectively reproduce astrocytic and neuronal features [27,28,29,57], these findings should be further confirmed in primary cell cultures. Although further efforts should be made to strengthen the involvement of neurotrophin signaling in brain cholesterol homeostasis, this work demonstrates for the first time that NGF may indirectly exert neuroprotection by influencing glial cholesterol metabolism.

## 4. Material and Methods

### 4.1. Cell Culture

N1E-115 neuroblastoma cells and U373 cells were cultured at 5% CO_2_ in DMEM at high glucose (Merck Life Science, Milan, Italy), with 10% (*v*/*v*) fetal bovine serum (FBS) (Merck Life Science, Milan, Italy), and added with penicillin/streptomycin solution. 

To induce neuronal differentiation, N1E-115 cells were seeded at 30% confluency and were switched to DMEM with 0.5% FBS for 96 h. 

Astrocyte-conditioned medium was prepared as previously reported [58]. Briefly, U373 were seeded at 70% confluency in 6-wells and maintained in DMEM with 10% FBS for 24 h. Cells were washed 3 times with PBS (Merck Life Science, Milan, Italy) and incubated with serum-free DMEM for 24 h. The medium was collected, centrifuged to remove cell debris, and immediately used for conditioned-medium experiments or kept at 80 °C for subsequent use. In all the experiments, N1E-115 cells were cultured in U373-conditioned medium diluted with fresh DMEM (dilution ratio 1:1), with a final concentration of 0.5% FBS.

Astrocyte–neuron co-cultures were set up according to the protocol described by Ioannou and colleagues, with modifications [59]. Paraffin spacers were pressed onto coverslips to assure adherence; we used spacers on neuronal coverslips to efficiently prevent mechanical damage when the cells were placed facing each other. Coverslips were then sterilized in ethanol and coated with poly-D-lysine (Merck Life Science, Milan, Italy). N1E-115 were seeded on the coated coverslips at the desired density and, once ready for the co-culture, sterile forceps were used to lift the coverslips with N1E-115 neurons and place them face-down into 6-wells seeded with U373 astrocytes.

Pre-treatments on N1E-115 cells were carried out by administering 0.1 µM of rotenone (Sigma-Aldrich, Milan, Italy, R8875) for 16 h. Control cells received DMSO (dilution 1:1000 in cell culture medium) as vehicle. U373 cells were treated with NGF (Alomone Labs, Jerusalem, Israel, N-245) at the dose of 100 ng/mL for 48 h in all the experiments.

### 4.2. Lysate Preparation and Western Blot Analysis

U373 cells were sonicated (duty cycle 20%, output 3) in sample buffer (0.125 M Tris-HCl containing 10% SDS, protease inhibitor cocktail, pH 6.8) for 30 s in order to obtain a total lysate, as previously described [60,61]. Laemmli buffer was added, and samples were denatured at 95 °C for 5 min. Protein extracts (twenty micrograms of proteins) were resolved on SDS–PAGE, and transfer onto nitrocellulose membrane was performed by using a trans-blot turbo transfer system (Biorad Laboratories, Milan, Italy), as previously reported [62]. Subsequently, the membrane was incubated at room temperature for 1 h with 5% no-fat dry milk in Tris-buffered saline (25 mM Tris-HCl, 138 mM NaCl, 27 mM KCl, 0.05% Tween-20, pH 6.8) and probed overnight at 4 °C with the following primary antibodies: anti-p75NTR (Santa Cruz Biotechnology, Dallas, TX, USA, sc-271708, dilution 1:500), anti-TrkA (Santa Cruz Biotechnology, Dallas, TX, USA, sc-118, dilution 1:500), anti-SREBP-1 (Santa Cruz Biotechnology, Dallas, TX, USA, sc-8984, dilution 1:1000), anti-SREBP-2 (Abcam, Cambridge, UK, ab30682, dilution 1:1000), anti-HMGCR (Abcam, Cambridge, UK, ab242315, dilution 1:1000), anti-LDLr (Abcam, Cambridge, UK, ab30532, dilution 1:500), anti-NPC1 (Novus Biologicals, Centennial, CO, USA, NB400-148, dilution 1:3000), anti-ABCA1 (Santa Cruz Biotechnology, Dallas, TX, USA, sc-58219, dilution 1:400), anti-ApoE (Santa Cruz Biotechnology, Dallas, TX, USA, sc-53570, dilution 1:400), anti-vinculin (Sigma-Aldrich, Milan, Italy, V9264, dilution 1:10,000), and anti-β-actin (Santa Cruz Biotechnology, Dallas, TX, USA, sc-47778, dilution 1:10,000). Membranes were successively incubated for 1 h at room temperature with HRP-conjugated secondary anti-mouse or anti-rabbit antibodies (Bio-Rad Laboratories, Milan, Italy). Protein-bound antibodies were visualized by clarity ECL Western blotting (Bio-Rad Laboratories, Milan, Italy, #1705061), and chemiluminescence was registered through the ChemiDoc MP system (Bio-Rad Laboratories, Milan, Italy). Densitometric analysis derived from Western blots was then carried out by using ImageJ version 1.52t (National Institutes of Health, Bethesda, MD, USA) software for Windows. Vinculin or β-actin were used as housekeeping proteins, which served as internal controls for protein loading. Densitometric calculations were obtained as arbitrary units, derived from the ratio between the intensity of protein band and the respective housekeeping protein.

### 4.3. Oil Red O Staining

U373 cells were seeded on poly-L-lysine (Sigma-Aldrich, P6282-5MG)-coated coverslips in 6-well plates, and Oil red O staining was performed as previously reported [60]. Briefly, cells were fixed in paraformaldehyde (4% solution) for 10 min and gently rinsed three times with PBS. Fixed cells were incubated with 60% isopropanol for 5 min and then washed with distilled water. Subsequently, cells were probed with 1 mL of Oil Red O working solution (Sigma-Aldrich, O1391-250ML) for 15 min at room temperature by putting the 6-well plate onto an orbital rotator shaker. After the incubation with the staining solution, wells were washed three times with distilled water to eliminate the excess stain. Coverslips were finally mounted with Fluoroshield mounting medium (Sigma-Aldrich, F6182), and Oil red O autofluorescence was visualized via confocal microscopy (TCS SP8; Leica, Wetzlar, Germany). Images were captured using Leica TCS SP8 equipped with a 63× magnification and Leica LAS X Software (Milan, Italy). Quantification of Oil red O staining was performed through ImageJ software for Windows and calculated as mean fluorescence intensity per cell area.

### 4.4. Filipin Staining

Filipin staining was carried out using Filipin complex (Sigma-Aldrich, F9765). Filipin stock solution (10 mg/mL in PBS) was always prepared immediately before use. Cells were fixed in paraformaldehyde (4% solution) for 10 min and washed with PBS. U373 were then stained with 1 mL Filipin working solution (0.05 mg/mL in PBS) for 1 h in the dark, at room temperature. Subsequently, cells were washed three times with PBS, and coverslips were mounted with Fluoroshield mounting medium and immediately analyzed through confocal microscopy using a UV filter set (340–380 nm excitation). Images were acquired at 40X magnification. Filipin quantification was calculated as mean fluorescence intensity per cell area by using ImageJ software for Windows.

### 4.5. Cholesterol Quantification

Cholesterol quantification was performed by using a colorimetric assay (Cholesterol Quantitation Kit, MAK043, Sigma-Aldrich, Milan, Italy) following the manufacturer’s instructions.

### 4.6. ELISA

ApoE levels in culture medium were assessed by using Human Apolipoprotein E ELISA Kit (Abcam, Cambridge, UK, ab108813), according to the manufacturer’s instructions.

### 4.7. Immunofluorescence

Immunofluorescence of U373 cells was executed as previously described [60]. U373 were fixed in paraformaldehyde (4% in PBS) and probed overnight with primary antibodies: anti-p75NTR (Santa Cruz Biotechnology, Dallas, TX, USA, sc-271708, dilution 1:100), and anti-TrkA (Santa Cruz Biotechnology, Dallas, TX, USA, sc-118, dilution 1:100). Subsequently, cells were incubated for 1 h with goat anti-mouse secondary antibody Alexa Fluor 555 (ThermoFisher Scientific, Waltham, MA, USA, A28180) and with goat anti-rabbit secondary antibody Alexa Fluor 488 (ThermoFisher Scientific, Waltham, MA, USA, A27034). DAPI staining was performed to visualize nuclei, and coverslips were finally mounted with Fluoroshield mounting medium. Samples were analyzed via confocal microscopy, as described above.

### 4.8. ApoE Silencing

ApoE mRNA silencing was performed on U373 cells using ApoE siRNA (Santa Cruz Biotechnology, Dallas, TX, USA, sc-29708) according to the manufacturer’s instructions. The transfection was set up in a 6-well tissue culture plate where 100,000 cells per well were seeded in DMEM antibiotic-free growth medium supplemented with 10% of FBS for 24 h. For each well, siRNA Transfection Medium (Santa Cruz Biotechnology, Dallas, TX, USA, sc-36868) was added to siRNA Transfection Reaction solution, prepared using siRNA duplex and siRNA Transfection Reagent (Santa Cruz Biotechnology, Dallas, TX, USA, sc-29528). Cells were then washed once with siRNA Transfection Medium and covered with the previously made transfection mixture. Subsequently, the culture plate was incubated for 7 h at 37 °C in a 5% CO_2_ incubator. DMEM growth medium with 20% of FBS and antibiotics (2-fold the normal concentration) was added to the transfection mixture in each well with a 1:1 ratio. Cells were incubated for 18 h, and then the medium was discharged and replaced with fresh normal growth medium; 24 h after this last step, cells were ready to be used for further experiments. The efficiency of ApoE silencing was assessed by RT-qPCR in U373 cells and by ELISA in the cell supernatant; ApoE siRNA efficiently prevented ApoE induction by NGF if compared to scramble siRNA (ApoE mRNA: 83% decrease; ApoE ELISA: 78% decrease). Notably, ApoE expression upon siRNA silencing was lower than the basal expression levels observed in control U373 (ApoE mRNA: 72% decrease; ApoE ELISA: 58% decrease) (Figure S3A,B).

### 4.9. RNA Extraction and Real-Time PCR

mRNA analysis was performed as previously reported [22,63]. Briefly, total RNA from U373 cells was extracted with TRI Reagent (Merck Life Science, Milan, Italy) according to the manufacturer’s instructions. DNAse treatment (Ambion, Thermo Fisher Scientific, Milan, Italy) was performed, and then RNA was purified using an RNA clean up Kit (Zymo, Italy). RNA was successively reverse transcribed to cDNA through a High-Capacity cDNA Reverse Transcription Cell Kit (Applied Bio-System, Foster City, CA, USA) and used for qPCR analysis. Primers used in qRT-PCR for *apoE* were: forward 5′-GGGTCGCTTTTGGGATTACCTG-3′ and reverse 5′-CAACTCCTTCATGGTCTCGTCC-3′. Primers used in qRT-PCR for *gapdh* (chosen as reference gene) were: forward 5′-GTCTCCTCTGACTTCAACAGCG-3′ and reverse 5′-ACCACCCTGTTGCTGTAGCCAA-3′. The production of the correct amplicon was assessed by the evaluation of the melting curve. Each biological sample was run in triplicate with SYBR green IQ reagent (Bio-Rad Laboratories, Milan, Italy) and using the CFX Connect detection system (Bio-Rad Laboratories, Milan, Italy). 

### 4.10. Quantitative Assessment of Neuronal Morphology

Neuronal morphology in N1E-115 cells was estimated by capturing images with an inverted microscope. Morphometric analysis was carried out through ImageJ software for Windows, and the number of total cells (expressed as percentage variation of the control), average neurite length (expressed as percentage of the control) and neurite-bearing cells were estimated. For neurite-bearing cells, the percentage was calculated by dividing the number of differentiated cells with the total number of cells per field. N1E-115 were defined differentiated when they had at least one neurite with length equal to or greater than the diameter of the soma. Morphological evaluation was performed on at least five independent experiments, in which at least three images were analyzed.

### 4.11. Statistical Analysis

All the results presented in this study were expressed as mean ± SD (standard deviation). Normal distribution of the data was checked by using the Shapiro–Wilk test. When two experimental groups were compared, unpaired Student’s *t* tests were applied. However, when three or more groups were compared, one-way analysis of variance (ANOVA) followed by Tukey’s post hoc test was employed. Concerning the experiments with rotenone, where two variables were present, two-way ANOVA followed by a Bonferroni post test was used. Values of *p* < 0.05 were considered to be statistically different. Statistical analysis was carried out using GraphPad Prism 5 (GraphPad, La Jolla, CA, USA) for Windows.

## Data Availability

Not applicable.

## Supplementary Materials

The following supporting information can be downloaded at: https://www.mdpi.com/article/10.3390/ijms23094842/s1.
