# Supplementary material for: NGF Modulates Cholesterol Metabolism and Stimulates ApoE Secretion in Glial Cells Conferring Neuroprotection against Oxidative Stress
Source: Int J Mol Sci. 2022 Apr 27;23(9):4842. doi: 10.3390/ijms23094842 (PMC9100774; doi:10.3390/ijms23094842)
Supplement: Supplementary file 1 [file ijms-23-04842-s001.zip › ijms-1707204-supplementary.pdf]

# NGF modulates cholesterol metabolism and stimulates ApoE secretion in glial cells conferring neuroprotection against oxidative stress

Mayra Colardo [1], Michele Petraroia [1], Letizia Lerza [1], Daniele Pensabene [2], Noemi Martella [1], Valentina Pallottini [2] and Marco Segatto [1,*]

[1] Department of Biosciences and Territory, University of Molise, Contrada Fonte Lappone, 86090 Pesche, Italy; m.colardo@studenti.unimol.it (M.C.); m.petraroia2@studenti.unimol.it (M.P.); n.martella@studenti.unimol.it (N.M.); l.lerza@studenti.unimol.it (L.L.); marco.segatto@unimol.it (M.S.)

[2] Department of Science, University Roma Tre, Viale Marconi 446, 00146 Rome, Italy; dan.pensabene@stud.uniroma3.it (D.P.); valentina.pallottini@uniroma3.it (V.P.)

[3] Neuroendocrinology Metabolism and Neuropharmacology Unit, IRCSS Fondazione Santa Lucia, Via del Fosso Fiorano 64, 00143 Rome, Italy.

* Correspondence: marco.segatto@unimol.it (M.S.)

## SUPPLEMENTARY FIGURES

### Figure S1

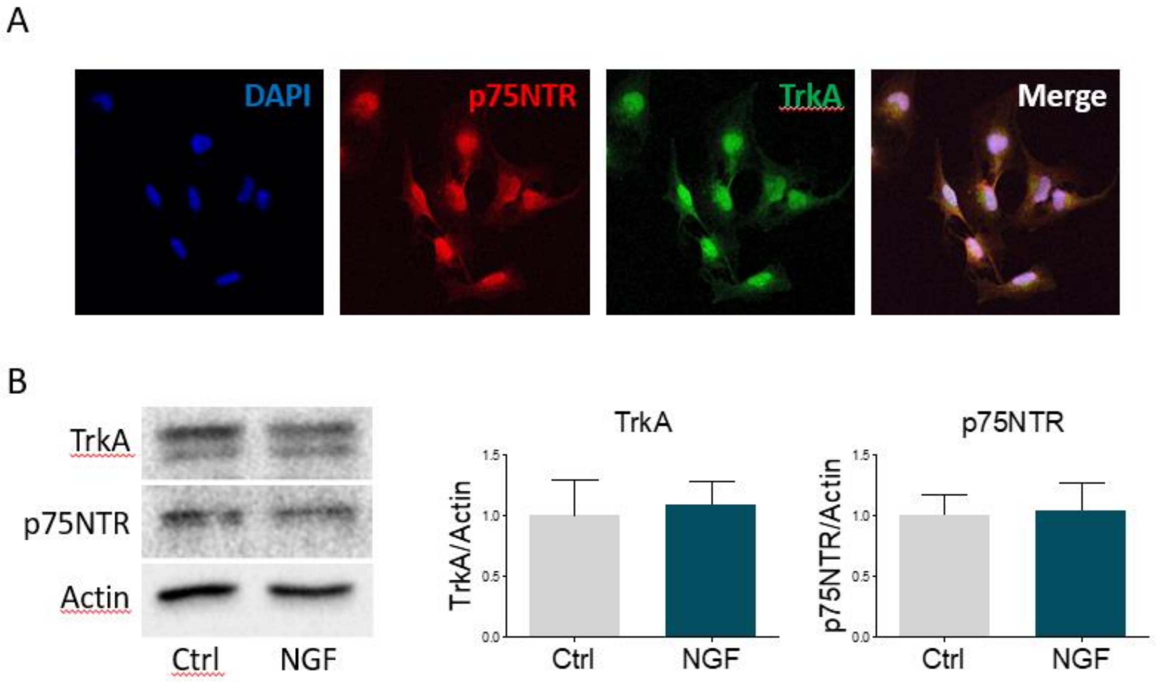


**Figure S1.** TrkA and p75NTR are expressed in U373 cells. (**A**) U373 cells were fixed in 4% PFA and stained with antibodies against p75NTR (red) and TrkA (green). DAPI was used to counterstain nuclei. (**B**) Representative Western blot and densitometric analysis of TrkA and p75NTR in U373 cells treated with vehicle (Ctrl) and NGF (100 ng/ml) for 48 hours. n= 3 different experiment. Actin was used as loading control. Data represent means ± SD. Statistical analysis was assessed by using unpaired Student's t test.

### Figure S2

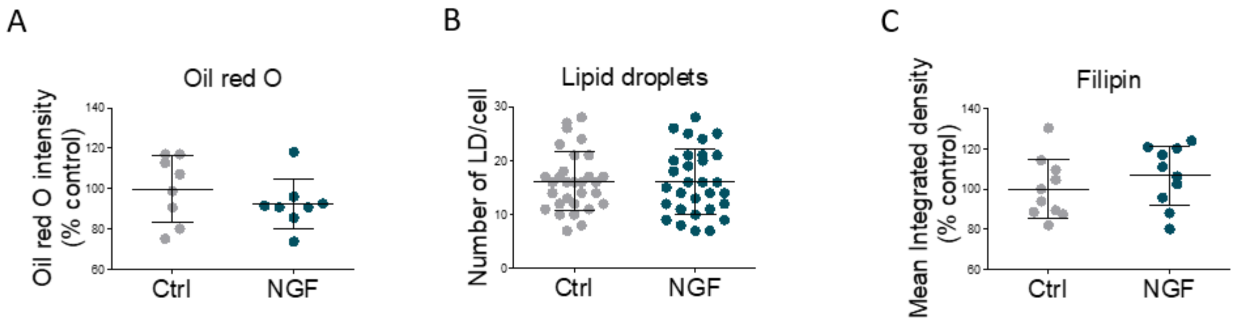


**Figure S2.** Quantification of Oilr red O, number of lipid droplets, and filipin staining in ctrl- and NGF treated U373 cells. (**A**) Oil red O intensity (percentage to control) in U373 cells treated with vehicle (Ctrl) and NGF (100 ng/ml) for 48 hours. n= 3 different experiments. (**B**) Analysis of the number of lipid droplets in U373 cells, treated as described in (A). n= 3 different experiments. (**C**) Quantification of the mean integrated density of filipin staining performed on U373 cells treated with vehicle and NGF for 48 hours. n= 3 different experiments. Data represent means ± SD. Statistical analysis was performed by using unpaired Student's t test.

**Figure S3**

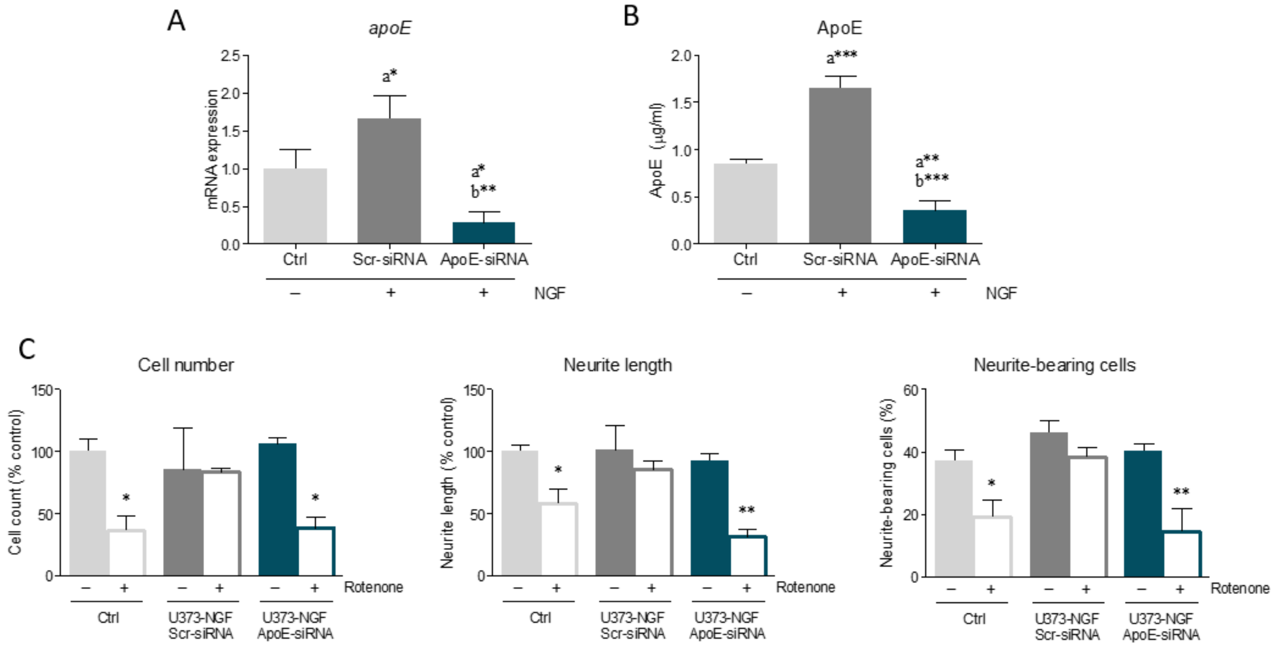


**Figure S3.** Validation of *apoE* silencing and its impact on neuroprotection. (**A**) qRT-PCR analysis of transcript levels for *apoE* in control-U373 (Ctrl), control-siRNA U373 (Scr-siRNA) and in ApoE-silenced U373 (ApoE-siRNA) in presence (+) or in absence (-) of NGF (100 ng/ml). n= 3 different experiments. "a" indicates statistical significance vs Ctrl group; "b" indicates statistical significance vs Scr-siRNA group. (**B**) Quantification of ApoE levels (µg/ml) by ELISA assay in culture medium derived from control-U373 (Ctrl), control-siRNA U373 (Scr-siRNA) and in ApoE-silenced U373 (ApoE-siRNA) with (+) or without (-) NGF treatment. n= 3 different experiments. (**C**) Quantitative assessment of neuronal morphology of N1E-115 cells, previously treated (+) or not (-) with rotenone (0.1 µM) for 16 hours, cultured in fresh DMEM (Ctrl), in conditioned medium derived from control-siRNA NGF-treated U373 (U373-NGF Scr-siRNA) and in conditioned medium derived from ApoE-silenced NGF-treated U373 (U373-NGF ApoE siRNA). n= 3 different experiments. Data represent means ± SD. Statistical analysis was assessed by using one-way ANOVA, followed by Tukey's post hoc. * $p < 0.05$, ** $p < 0.01$, *** $p < 0.001$. "a" indicates statistical significance vs Ctrl group; "b" indicates statistical significance vs Scr-siRNA group.
